# 7-(3-Nitro­phen­yl)-9,10-dihydro-7*H*-benzo[*h*]cyclo­penta­[*b*]quinolin-8(11*H*)-one

**DOI:** 10.1107/S1600536811043546

**Published:** 2011-10-29

**Authors:** Tuanjie Li, Honghong Zhang

**Affiliations:** a School of Chemistry and Chemical Engineering, Jiangsu Key Laboratory of Green Synthetic Chemistry for Functional Materials, Xuzhou Normal University, Xuzhou, Jiangsu 221116, People’s Republic of China

## Abstract

In the title compound, C_22_H_16_N_2_O_3_, the naphthalene ring, the 1,4-dihydro­pyridine ring and the cyclo­pent-2-enone ring are nearly coplanar, with the dihedral angles between the neighbouring rings being 1.93 (11) and 2.30 (9)°, respectively. The benzene ring group at position 7 and the 1,4-dihydro­pyridine ring form a dihedral angle of 78.75 (4)°. Inter­molecular N—H⋯O hydrogen bonds and C—H⋯π inter­actions stabilize the crystal packing.

## Related literature

For the medicinal use of 1,4-dihydro­pyridine derivatives, see: Zheng *et al.* (2011 ▶); Ginsberg & Kummer (2011 ▶); Nadaraj *et al.* (2009 ▶); Husson *et al.* (2011 ▶). For the preparation of the title compound, see: Heravi *et al.* (2010 ▶).
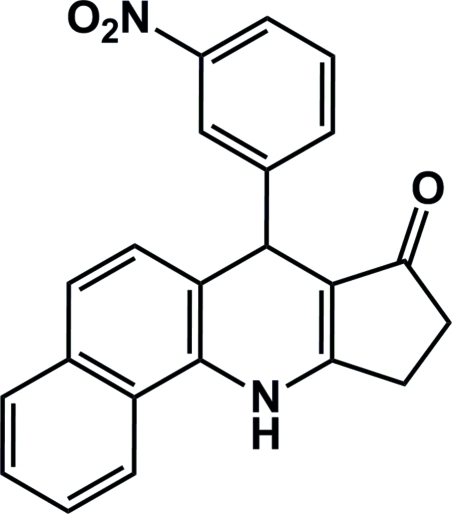


## Experimental

### 

#### Crystal data

C_22_H_16_N_2_O_3_
*M* *_r_* = 356.37
Monoclinic, 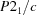

*a* = 10.256 (1) Å
*b* = 13.7570 (14) Å
*c* = 11.9830 (12) Å
β = 104.827 (5)°
*V* = 1634.4 (3) Å^3^
*Z* = 4
Mo *K*α radiation
μ = 0.10 mm^−1^
*T* = 113 K
0.24 × 0.20 × 0.18 mm

#### Data collection

Rigaku Saturn724 CCD diffractometer
Absorption correction: multi-scan (*CrystalClearSM Expert*; Rigaku/MSC, 2009 ▶) *T*_min_ = 0.977, *T*_max_ = 0.983
16937 measured reflections
3897 independent reflections
3094 reflections with *I* > 2σ(*I*)
*R*_int_ = 0.046

#### Refinement

*R*[*F* ^2^ > 2σ(*F* ^2^)] = 0.055
*wR*(*F* ^2^) = 0.130
*S* = 1.12
3897 reflections
248 parameters
H atoms treated by a mixture of independent and constrained refinement
Δρ_max_ = 0.27 e Å^−3^
Δρ_min_ = −0.21 e Å^−3^

### 

Data collection: *CrystalClearSM Expert* (Rigaku/MSC, 2009 ▶); cell refinement: *CrystalClearSM Expert*; data reduction: *CrystalClearSM Expert*; program(s) used to solve structure: *SHELXS97* (Sheldrick, 2008 ▶); program(s) used to refine structure: *SHELXL97* (Sheldrick, 2008 ▶); molecular graphics: *SHELXTL* (Sheldrick, 2008 ▶); software used to prepare material for publication: *SHELXTL*.

## Supplementary Material

Crystal structure: contains datablock(s) I, global. DOI: 10.1107/S1600536811043546/hg5109sup1.cif

Structure factors: contains datablock(s) I. DOI: 10.1107/S1600536811043546/hg5109Isup2.hkl

Supplementary material file. DOI: 10.1107/S1600536811043546/hg5109Isup3.cml

Additional supplementary materials:  crystallographic information; 3D view; checkCIF report

## Figures and Tables

**Table 1:** Hydrogen-bond geometry (Å, °) *Cg* is the centroid of the ring of C11–C16.

*D*—H⋯*A*	*D*—H	H⋯*A*	*D*⋯*A*	*D*—H⋯*A*
N1—H1⋯O1^i^	0.881 (19)	2.07 (2)	2.9307 (17)	165.9 (18)
C21—H21⋯*Cg*^ii^	0.95	2.69	3.5090 (19)	145

Symmetry codes: (i) 
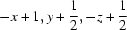
; (ii) 
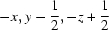
.

**Fig. 1.**
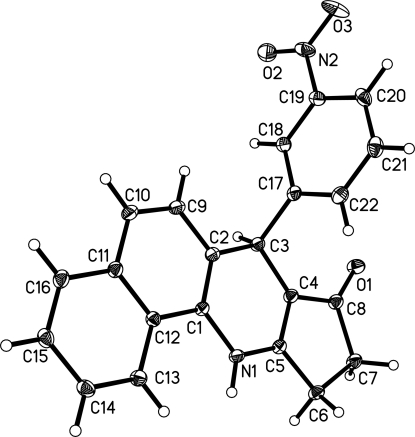
The structure of (I), showing 30% probability displacement ellipsoids and the atom-numbering scheme. Cg is the centroid of the ring of C11/C12/C13/C14/C15/C16.

**Fig. 2.**
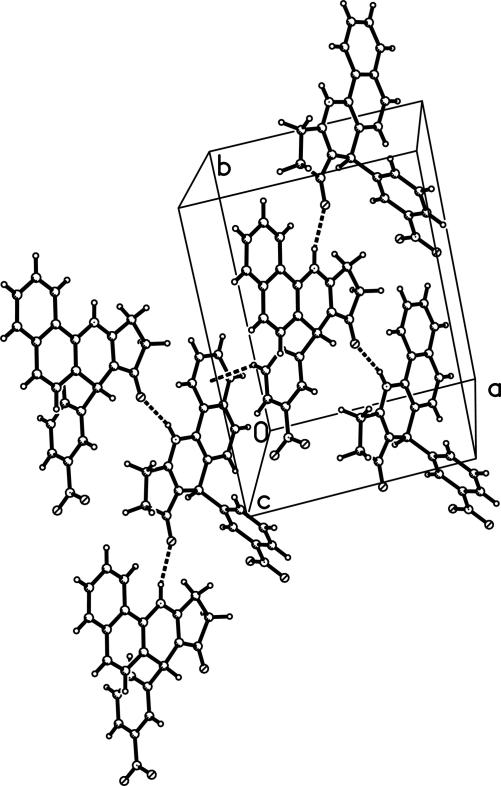
The packing diagram of (I), Hydrogen bond represented by the dashed line.

## supplementary crystallographic information

### Comment

The 1,4-dihydropyridine (1,4-DHP) derivatives exhibits various bioactivities, including sedative-hypnotic activity (Zheng *et al.* 2011), inhibition of the α 4-integrin-paxillin interaction (Ginsberg *et al.* 2011), anti-microbial activities (Nadaraj *et al.* 2009),and antitumor activity (Husson *et al.* 2011). These reports inspired us to study the relationship between their structures and activities. During the synthesis of 1,4-dihydropyridine (1,4-DHP) derivatives, the title compound, (I) was isolated and its structure was determined by X-ray diffraction. Herein we report its crystal structure.

In the molecular structure (Fig. 1), the naphthalene ring, the 1,4-dihydropyridine ring and the cyclopent-2-enone ring adopt planar conformations with RMS of 0.0201 Å, 0.0235 Å and 0.0058 Å, respectively. The largest deviation of these rings are 0.033 (1) Å(C2), 0.038 (1) Å(C3), 0.008 (1) Å(C5), respectively. The fused ring system is almost coplanar, for the dihedral angle between the neighboring rings are 1.93 (0.11) ° and 2.30 (9)° respectively. The planar 3-nitrophenyl ring at position 7 and the 1,4-dihydropyridine ring forms a dihedral angle of 78.75 (4)°. The crystal packing is stablized by the intermolecular N—H···O hydrogen bond and C—H···π interactions (Fig. 2, Table 1).

### Experimental

The title compound was synthesized according to the procedure (Heravi *et al.* 2010). A round-bottomed flask was charged with 3-nitrobenzaldehyde (1 mmol), cyclopentane-1,3-dione (1 mmol), 1-naphtylamine (1 mmol), acetic acid (5 ml), and H_6_P_2_W_18_O_62.18_H_2_O (0.01 mmol). The reaction mixture was stirred until completion (monitored by TLC). Then the mixture was poured into ice water. The precipitated products were separated by filtration, washed with water, recrystallized in a dimethylformamide-ethanol (DMF-EtOH) solution. The recrystallization gave single-crystals suitable for X-ray diffraction.

### Refinement

The hydrogen atom bonded to the nitrogen atom was positioned from a Fourier difference map refined freely. All other H atoms were placed in calculated positions, with C—H = 0.95 Å, 0.99Å or 1.00Å and included in the final cycles of refinement using a riding model, with U_iso_(H) = 1.2U_eq_(parent atom).

### Crystal data

**Table:**

C_22_H_16_N_2_O_3_	*F*(000) = 744
*M**_r_* = 356.37	*D*_x_ = 1.448 Mg m^−^^3^
Monoclinic, *P*2_1_/*c*	Mo *K*α radiation, λ = 0.71075 Å
Hall symbol: -P 2ybc	Cell parameters from 4977 reflections
*a* = 10.256 (1) Å	θ = 1.8–28.0°
*b* = 13.7570 (14) Å	µ = 0.10 mm^−^^1^
*c* = 11.9830 (12) Å	*T* = 113 K
β = 104.827 (5)°	Prism, colorless
*V* = 1634.4 (3) Å^3^	0.24 × 0.20 × 0.18 mm
*Z* = 4	

### Data collection

**Table:**

Rigaku Saturn724 CCD diffractometer	3897 independent reflections
Radiation source: rotating anode	3094 reflections with *I* > 2σ(*I*)
multilayer	*R*_int_ = 0.046
Detector resolution: 14.222 pixels mm^-1^	θ_max_ = 27.9°, θ_min_ = 2.1°
ω scans	*h* = −13→13
Absorption correction: multi-scan (CrystalClearSM Expert; Rigaku/MSC, 2009)	*k* = −18→16
*T*_min_ = 0.977, *T*_max_ = 0.983	*l* = −14→15
16937 measured reflections	

### Refinement

**Table:**

Refinement on *F*^2^	Primary atom site location: structure-invariant direct methods
Least-squares matrix: full	Secondary atom site location: difference Fourier map
*R*[*F*^2^ > 2σ(*F*^2^)] = 0.055	Hydrogen site location: inferred from neighbouring sites
*wR*(*F*^2^) = 0.130	H atoms treated by a mixture of independent and constrained refinement
*S* = 1.12	*w* = 1/[σ^2^(*F*_o_^2^) + (0.0585*P*)^2^ + 0.0399*P*] where *P* = (*F*_o_^2^ + 2*F*_c_^2^)/3
3897 reflections	(Δ/σ)_max_ < 0.001
248 parameters	Δρ_max_ = 0.27 e Å^−^^3^
0 restraints	Δρ_min_ = −0.21 e Å^−^^3^

### Special details

**Table:**

Geometry. All e.s.d.'s (except the e.s.d. in the dihedral angle between two l.s. planes) are estimated using the full covariance matrix. The cell e.s.d.'s are taken into account individually in the estimation of e.s.d.'s in distances, angles and torsion angles; correlations between e.s.d.'s in cell parameters are only used when they are defined by crystal symmetry. An approximate (isotropic) treatment of cell e.s.d.'s is used for estimating e.s.d.'s involving l.s. planes.
Refinement. Refinement of *F*^2^ against ALL reflections. The weighted *R*-factor *wR* and goodness of fit *S* are based on *F*^2^, conventional *R*-factors *R* are based on *F*, with *F* set to zero for negative *F*^2^. The threshold expression of *F*^2^ > σ(*F*^2^) is used only for calculating *R*-factors(gt) *etc*. and is not relevant to the choice of reflections for refinement. *R*-factors based on *F*^2^ are statistically about twice as large as those based on *F*, and *R*– factors based on ALL data will be even larger.

### Fractional atomic coordinates and isotropic or equivalent isotropic displacement parameters (Å^2^)

**Table:**

	*x*	*y*	*z*	*U*_iso_*/*U*_eq_	
O1	0.49879 (12)	0.27956 (8)	0.21675 (10)	0.0302 (3)	
O2	0.22400 (14)	0.05360 (8)	0.50038 (12)	0.0396 (3)	
O3	0.05702 (14)	0.00451 (9)	0.36064 (13)	0.0555 (5)	
N1	0.40366 (13)	0.59115 (10)	0.34262 (12)	0.0204 (3)	
N2	0.14286 (15)	0.06518 (10)	0.40583 (15)	0.0333 (4)	
C1	0.32624 (14)	0.56590 (10)	0.42002 (13)	0.0182 (3)	
C2	0.30280 (15)	0.46920 (11)	0.44074 (14)	0.0210 (3)	
C3	0.36250 (15)	0.38501 (11)	0.38630 (13)	0.0205 (3)	
H3	0.4315	0.3524	0.4494	0.025*	
C4	0.43418 (15)	0.42402 (11)	0.30100 (14)	0.0209 (3)	
C5	0.45220 (14)	0.52071 (11)	0.28614 (13)	0.0192 (3)	
C6	0.53292 (16)	0.54216 (11)	0.20092 (14)	0.0237 (4)	
H6A	0.6177	0.5766	0.2382	0.028*	
H6B	0.4805	0.5820	0.1360	0.028*	
C7	0.56175 (17)	0.44056 (11)	0.15962 (15)	0.0249 (4)	
H7A	0.5219	0.4336	0.0756	0.030*	
H7B	0.6601	0.4290	0.1761	0.030*	
C8	0.49657 (16)	0.36910 (12)	0.22699 (14)	0.0232 (4)	
C9	0.22432 (16)	0.44723 (11)	0.51891 (14)	0.0261 (4)	
H9	0.2063	0.3811	0.5322	0.031*	
C10	0.17360 (16)	0.51798 (11)	0.57591 (14)	0.0269 (4)	
H10	0.1210	0.5004	0.6275	0.032*	
C11	0.19893 (15)	0.61746 (11)	0.55856 (13)	0.0212 (3)	
C12	0.27526 (14)	0.64231 (10)	0.47886 (13)	0.0191 (3)	
C13	0.29923 (16)	0.74235 (11)	0.46203 (14)	0.0226 (4)	
H13	0.3481	0.7607	0.4079	0.027*	
C14	0.25244 (15)	0.81264 (12)	0.52334 (14)	0.0251 (4)	
H14	0.2696	0.8792	0.5112	0.030*	
C15	0.17967 (16)	0.78757 (12)	0.60362 (14)	0.0251 (4)	
H15	0.1493	0.8369	0.6464	0.030*	
C16	0.15242 (15)	0.69215 (12)	0.62036 (14)	0.0248 (4)	
H16	0.1018	0.6757	0.6739	0.030*	
C17	0.25581 (15)	0.30911 (11)	0.33354 (13)	0.0202 (3)	
C18	0.24757 (15)	0.22350 (11)	0.39195 (14)	0.0219 (4)	
H18	0.3082	0.2114	0.4650	0.026*	
C19	0.14954 (15)	0.15545 (11)	0.34252 (14)	0.0235 (4)	
C20	0.05978 (16)	0.16991 (12)	0.23582 (15)	0.0289 (4)	
H20	−0.0061	0.1223	0.2032	0.035*	
C21	0.06897 (17)	0.25572 (13)	0.17830 (15)	0.0307 (4)	
H21	0.0088	0.2675	0.1049	0.037*	
C22	0.16534 (16)	0.32482 (12)	0.22678 (14)	0.0264 (4)	
H22	0.1696	0.3837	0.1865	0.032*	
H1	0.4201 (19)	0.6518 (14)	0.3272 (16)	0.041 (6)*	

### Atomic displacement parameters (Å^2^)

**Table:**

	*U*^11^	*U*^22^	*U*^33^	*U*^12^	*U*^13^	*U*^23^
O1	0.0399 (7)	0.0183 (6)	0.0395 (8)	0.0010 (5)	0.0233 (6)	−0.0023 (5)
O2	0.0559 (8)	0.0246 (7)	0.0386 (8)	−0.0027 (6)	0.0127 (7)	0.0029 (6)
O3	0.0480 (8)	0.0283 (7)	0.0848 (12)	−0.0197 (6)	0.0072 (8)	−0.0018 (7)
N1	0.0254 (7)	0.0159 (6)	0.0234 (8)	−0.0002 (5)	0.0123 (6)	0.0007 (5)
N2	0.0336 (8)	0.0184 (7)	0.0514 (11)	−0.0038 (6)	0.0173 (8)	−0.0044 (7)
C1	0.0190 (7)	0.0197 (8)	0.0172 (8)	0.0000 (6)	0.0069 (6)	0.0007 (6)
C2	0.0241 (8)	0.0196 (8)	0.0208 (9)	−0.0011 (6)	0.0082 (7)	0.0002 (6)
C3	0.0240 (8)	0.0184 (8)	0.0207 (8)	0.0007 (6)	0.0086 (7)	0.0012 (6)
C4	0.0234 (7)	0.0194 (8)	0.0217 (9)	0.0008 (6)	0.0094 (7)	0.0007 (6)
C5	0.0200 (7)	0.0201 (8)	0.0183 (8)	0.0017 (6)	0.0066 (6)	0.0013 (6)
C6	0.0288 (8)	0.0211 (8)	0.0256 (9)	0.0012 (7)	0.0148 (7)	0.0019 (7)
C7	0.0299 (8)	0.0232 (8)	0.0259 (9)	0.0027 (7)	0.0150 (7)	0.0001 (7)
C8	0.0253 (8)	0.0229 (8)	0.0233 (9)	0.0011 (7)	0.0095 (7)	0.0001 (7)
C9	0.0347 (9)	0.0199 (8)	0.0287 (10)	−0.0043 (7)	0.0172 (8)	−0.0005 (7)
C10	0.0340 (9)	0.0246 (9)	0.0277 (10)	−0.0037 (7)	0.0182 (8)	−0.0013 (7)
C11	0.0211 (7)	0.0230 (8)	0.0207 (9)	0.0005 (6)	0.0074 (7)	−0.0013 (7)
C12	0.0189 (7)	0.0188 (8)	0.0199 (8)	0.0008 (6)	0.0056 (6)	−0.0011 (6)
C13	0.0218 (8)	0.0215 (8)	0.0266 (9)	0.0005 (6)	0.0101 (7)	−0.0008 (7)
C14	0.0260 (8)	0.0188 (8)	0.0318 (10)	0.0004 (6)	0.0098 (7)	−0.0031 (7)
C15	0.0240 (8)	0.0245 (8)	0.0283 (10)	0.0013 (7)	0.0093 (7)	−0.0069 (7)
C16	0.0233 (8)	0.0295 (9)	0.0236 (9)	0.0005 (7)	0.0099 (7)	−0.0032 (7)
C17	0.0222 (8)	0.0189 (8)	0.0222 (9)	0.0031 (6)	0.0110 (7)	−0.0018 (6)
C18	0.0230 (8)	0.0195 (8)	0.0246 (9)	0.0018 (6)	0.0085 (7)	−0.0012 (6)
C19	0.0235 (8)	0.0177 (8)	0.0325 (10)	0.0001 (6)	0.0129 (7)	−0.0045 (7)
C20	0.0226 (8)	0.0311 (9)	0.0345 (10)	−0.0027 (7)	0.0101 (8)	−0.0149 (8)
C21	0.0246 (8)	0.0416 (11)	0.0248 (10)	0.0045 (8)	0.0043 (7)	−0.0041 (8)
C22	0.0257 (8)	0.0294 (9)	0.0261 (9)	0.0039 (7)	0.0099 (7)	0.0014 (7)

### Geometric parameters (Å, °)

**Table:**

O1—C8	1.2387 (18)	C9—H9	0.9500
O2—N2	1.2325 (18)	C10—C11	1.418 (2)
O3—N2	1.2333 (18)	C10—H10	0.9500
N1—C5	1.3481 (19)	C11—C16	1.419 (2)
N1—C1	1.4101 (19)	C11—C12	1.423 (2)
N1—H1	0.881 (19)	C12—C13	1.421 (2)
N2—C19	1.466 (2)	C13—C14	1.373 (2)
C1—C2	1.385 (2)	C13—H13	0.9500
C1—C12	1.437 (2)	C14—C15	1.403 (2)
C2—C9	1.415 (2)	C14—H14	0.9500
C2—C3	1.532 (2)	C15—C16	1.368 (2)
C3—C4	1.503 (2)	C15—H15	0.9500
C3—C17	1.528 (2)	C16—H16	0.9500
C3—H3	1.0000	C17—C18	1.384 (2)
C4—C5	1.361 (2)	C17—C22	1.392 (2)
C4—C8	1.434 (2)	C18—C19	1.390 (2)
C5—C6	1.499 (2)	C18—H18	0.9500
C6—C7	1.536 (2)	C19—C20	1.386 (2)
C6—H6A	0.9900	C20—C21	1.382 (2)
C6—H6B	0.9900	C20—H20	0.9500
C7—C8	1.530 (2)	C21—C22	1.387 (2)
C7—H7A	0.9900	C21—H21	0.9500
C7—H7B	0.9900	C22—H22	0.9500
C9—C10	1.366 (2)		
			
C5—N1—C1	119.70 (13)	C2—C9—H9	118.9
C5—N1—H1	117.4 (13)	C9—C10—C11	120.44 (15)
C1—N1—H1	122.9 (13)	C9—C10—H10	119.8
O2—N2—O3	123.67 (15)	C11—C10—H10	119.8
O2—N2—C19	118.37 (14)	C10—C11—C16	121.59 (15)
O3—N2—C19	117.95 (16)	C10—C11—C12	118.89 (14)
C2—C1—N1	120.48 (14)	C16—C11—C12	119.51 (14)
C2—C1—C12	120.86 (14)	C13—C12—C11	118.21 (14)
N1—C1—C12	118.65 (13)	C13—C12—C1	122.74 (14)
C1—C2—C9	118.55 (14)	C11—C12—C1	119.04 (13)
C1—C2—C3	122.89 (14)	C14—C13—C12	120.60 (15)
C9—C2—C3	118.52 (13)	C14—C13—H13	119.7
C4—C3—C17	112.70 (12)	C12—C13—H13	119.7
C4—C3—C2	109.77 (13)	C13—C14—C15	120.91 (15)
C17—C3—C2	111.79 (12)	C13—C14—H14	119.5
C4—C3—H3	107.4	C15—C14—H14	119.5
C17—C3—H3	107.4	C16—C15—C14	120.06 (15)
C2—C3—H3	107.4	C16—C15—H15	120.0
C5—C4—C8	109.70 (14)	C14—C15—H15	120.0
C5—C4—C3	122.99 (14)	C15—C16—C11	120.67 (15)
C8—C4—C3	127.29 (14)	C15—C16—H16	119.7
N1—C5—C4	123.85 (15)	C11—C16—H16	119.7
N1—C5—C6	122.65 (13)	C18—C17—C22	118.99 (14)
C4—C5—C6	113.49 (13)	C18—C17—C3	120.16 (13)
C5—C6—C7	103.04 (12)	C22—C17—C3	120.85 (14)
C5—C6—H6A	111.2	C17—C18—C19	119.25 (15)
C7—C6—H6A	111.2	C17—C18—H18	120.4
C5—C6—H6B	111.2	C19—C18—H18	120.4
C7—C6—H6B	111.2	C20—C19—C18	122.26 (15)
H6A—C6—H6B	109.1	C20—C19—N2	119.42 (15)
C8—C7—C6	105.57 (13)	C18—C19—N2	118.32 (15)
C8—C7—H7A	110.6	C21—C20—C19	117.95 (15)
C6—C7—H7A	110.6	C21—C20—H20	121.0
C8—C7—H7B	110.6	C19—C20—H20	121.0
C6—C7—H7B	110.6	C20—C21—C22	120.57 (16)
H7A—C7—H7B	108.8	C20—C21—H21	119.7
O1—C8—C4	127.41 (15)	C22—C21—H21	119.7
O1—C8—C7	124.41 (14)	C21—C22—C17	120.97 (16)
C4—C8—C7	108.18 (13)	C21—C22—H22	119.5
C10—C9—C2	122.19 (15)	C17—C22—H22	119.5
C10—C9—H9	118.9		
			
C5—N1—C1—C2	1.7 (2)	C10—C11—C12—C13	179.71 (13)
C5—N1—C1—C12	−179.79 (13)	C16—C11—C12—C13	−1.6 (2)
N1—C1—C2—C9	−179.88 (13)	C10—C11—C12—C1	−1.1 (2)
C12—C1—C2—C9	1.7 (2)	C16—C11—C12—C1	177.64 (13)
N1—C1—C2—C3	2.5 (2)	C2—C1—C12—C13	178.67 (14)
C12—C1—C2—C3	−175.93 (13)	N1—C1—C12—C13	0.2 (2)
C1—C2—C3—C4	−5.9 (2)	C2—C1—C12—C11	−0.5 (2)
C9—C2—C3—C4	176.43 (14)	N1—C1—C12—C11	−178.97 (13)
C1—C2—C3—C17	−131.76 (15)	C11—C12—C13—C14	1.5 (2)
C9—C2—C3—C17	50.61 (19)	C1—C12—C13—C14	−177.67 (14)
C17—C3—C4—C5	131.30 (15)	C12—C13—C14—C15	−0.2 (2)
C2—C3—C4—C5	6.0 (2)	C13—C14—C15—C16	−1.1 (2)
C17—C3—C4—C8	−50.8 (2)	C14—C15—C16—C11	1.0 (2)
C2—C3—C4—C8	−176.06 (14)	C10—C11—C16—C15	179.01 (14)
C1—N1—C5—C4	−1.8 (2)	C12—C11—C16—C15	0.3 (2)
C1—N1—C5—C6	178.78 (13)	C4—C3—C17—C18	134.40 (14)
C8—C4—C5—N1	179.14 (14)	C2—C3—C17—C18	−101.41 (16)
C3—C4—C5—N1	−2.6 (2)	C4—C3—C17—C22	−45.87 (19)
C8—C4—C5—C6	−1.35 (18)	C2—C3—C17—C22	78.32 (18)
C3—C4—C5—C6	176.90 (13)	C22—C17—C18—C19	0.1 (2)
N1—C5—C6—C7	−179.01 (14)	C3—C17—C18—C19	179.86 (13)
C4—C5—C6—C7	1.47 (17)	C17—C18—C19—C20	0.5 (2)
C5—C6—C7—C8	−0.98 (16)	C17—C18—C19—N2	179.86 (14)
C5—C4—C8—O1	−179.08 (15)	O2—N2—C19—C20	179.70 (15)
C3—C4—C8—O1	2.8 (3)	O3—N2—C19—C20	0.5 (2)
C5—C4—C8—C7	0.62 (18)	O2—N2—C19—C18	0.3 (2)
C3—C4—C8—C7	−177.54 (14)	O3—N2—C19—C18	−178.90 (15)
C6—C7—C8—O1	180.00 (15)	C18—C19—C20—C21	−0.5 (2)
C6—C7—C8—C4	0.29 (17)	N2—C19—C20—C21	−179.87 (14)
C1—C2—C9—C10	−1.3 (2)	C19—C20—C21—C22	−0.1 (2)
C3—C2—C9—C10	176.39 (14)	C20—C21—C22—C17	0.7 (2)
C2—C9—C10—C11	−0.3 (3)	C18—C17—C22—C21	−0.7 (2)
C9—C10—C11—C16	−177.23 (15)	C3—C17—C22—C21	179.55 (14)
C9—C10—C11—C12	1.5 (2)		

### Hydrogen-bond geometry (Å, °)

**Table:**

Cg is the centroid of the ring of C11/C12/C13/C14/C15/C16. [ok as edited?]

**Table:**

*D*—H···*A*	*D*—H	H···*A*	*D*···*A*	*D*—H···*A*
N1—H1···O1^i^	0.881 (19)	2.07 (2)	2.9307 (17)	165.9 (18)
C21—H21···Cg^ii^	0.95	2.69	3.5090 (19)	145.

Symmetry codes: (i) −*x*+1, *y*+1/2, −*z*+1/2; (ii) −*x*, *y*−1/2, −*z*+1/2.
